# A Reward-Based Behavioral Platform to Measure Neural Activity during Head-Fixed Behavior

**DOI:** 10.3389/fncel.2017.00156

**Published:** 2017-05-31

**Authors:** Andrew H. Micallef, Naoya Takahashi, Matthew E. Larkum, Lucy M. Palmer

**Affiliations:** ^1^The Florey Institute of Neuroscience and Mental Health, University of MelbourneMelbourne, VIC, Australia; ^2^Institute for Biology, Humboldt University of BerlinBerlin, Germany

**Keywords:** dendrites, behavior platform, methods, reward learning, two photon imaging

## Abstract

Understanding the neural computations that contribute to behavior requires recording from neurons while an animal is behaving. This is not an easy task as most subcellular recording techniques require absolute head stability. The Go/No-Go sensory task is a powerful decision-driven task that enables an animal to report a binary decision during head-fixation. Here we discuss how to set up an Ardunio and Python based platform system to control a Go/No-Go sensory behavior paradigm. Using an Arduino micro-controller and Python-based custom written program, a reward can be delivered to the animal depending on the decision reported. We discuss the various components required to build the behavioral apparatus that can control and report such a sensory stimulus paradigm. This system enables the end user to control the behavioral testing in real-time and therefore it provides a strong custom-made platform for probing the neural basis of behavior.

## Introduction

Neurons are the building blocks of behavior. Therefore, to understand the neural basis of behavior, we must record from individual neurons while an animal is active. This is no easy feat, however, as most techniques used to measure activity in a single neuron, such as patch-clamp electrophysiology and two-photon microscopy, require absolute stability of the preparation. Therefore, apart from a handful of studies using advanced techniques where subcellular neural recordings can be performed from animals physically moving through an environment, such as head-mounted two-photon microscopes (Helmchen et al., 2001; Sawinski et al., 2009) or electrophysiological microdrives (Holtmaat et al., 2009; Burgalossi et al., 2011), little is known about the single-cellular activity and even less about subcellular activity in behaving animals.

To unravel complex natural behavior, experimental paradigms are often simplified and designed for operant conditioning where certain behaviors are reinforced by the delivery of a reward. Typically, in a standard rodent reward-based behavioral test, the experimenter is able to gage the animal’s response to a given stimulus by monitoring movement/behavior. For example, in response to a rewardable stimulus/situation, an animal may be trained to nose-poke at a given location (Huber et al., 2008; Bussey et al., 2012; Nithianantharajah et al., 2013), press a lever (Lederle et al., 2011), or navigate through a particular sensory environment (van Praag et al., 2000; Znamenskiy and Zador, 2013). These behavioral tests require physical movement of the animal and therefore probing the associated neural activity typically involves gross recording techniques such as multi-unit electrophysiology (Epsztein et al., 2011; Stensola et al., 2012; Whitlock et al., 2012). To overcome this limitation, a head-fixed configuration can be imposed on behavioral tests where animals can still move to receive a stimulus and signal a response, however, their heads are stable to enable simultaneous neural recordings. In this configuration, the animal is affixed to a stationary apparatus with an implanted head-plate, and licking a sensor is often adopted as a behavioral readout of operant conditioning.

The Go/No-Go sensory task is a powerful decision-driven task that enables an animal to report a binary decision based on a received sensory stimulus. In “Go” trials, the subject is required to make a behavioral action (i.e., licking) in response to a target stimulus whereas in “No-Go” trials, the subject withholds a response. This sensory-based decision-making task has been used historically to address neural activity in monkeys (Mishkin and Pribram, 1955) and humans (Costantini and Hoving, 1973). More recently, various brain regions have been investigated during the Go/No-Go sensory task including the orbitofrontal cortex (Tremblay and Schultz, 2000) and nucleus basalis (Richardson and DeLong, 1990) in the monkey, and the barrel cortex (Petreanu et al., 2012; Xu et al., 2012; Chen et al., 2013; O’Connor et al., 2013), and motor cortex (Huber et al., 2012) in the mouse. The Go/No-Go task is advantageous over other decision-based sensory tasks as its relative simplicity makes it easily and reliably performed. Since head-fixed recording techniques are usually plagued with low-success rates, short behavioral tasks which can be learnt in a few sessions are preferred. Another popular decision-based sensory task is the two-alternative forced choice (2AFC) task which may involve more complicated neural computations than the Go/No-Go task, however, it takes considerably longer for animals to learn and execute the task. Although more training is generally required, 2AFC is advantageous as reporting of false-misses (which can occur during “No-Go” trials) is prevented by establishing two lick ports where the animal must report to either a left or right port according to a 2AFC detection task (Guo et al., 2014; Li et al., 2015).

To operate such a sensory task, a behavioral platform must be established which monitors the animals’ licking behavior, and subsequently delivers sensory stimuli and associated water rewards in real-time. The behavioral system described here is designed using an Arduino Uno Rev3 microcontroller, a low-cost, open source prototyping platform. Briefly, once loaded with a controller program, the Arduino operates with microsecond precision (16 MHz clock speed) to independently control TTL pulses at the output pins, read the inbuilt analog to digital converter, and perform two-way communications with the host computer for parameter update and data logging. Here, we discuss how to setup an Arduino-based behavioral platform for a Go/No-Go task with sensory stimulation. We discuss training paradigms associated with the behavior platform and we report on how this platform can be used to measure neural activity during reward-based behavior.

## Methods

### System Overview

**Figure 1:**
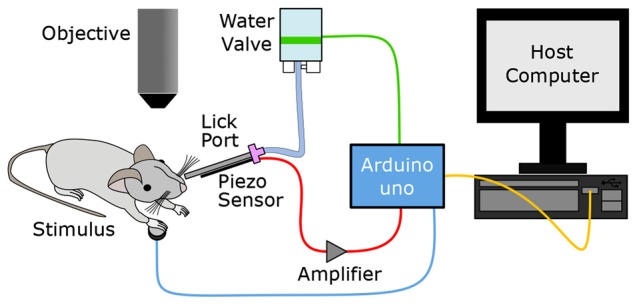
Overview of the Go/No-Go sensory task setup. An Arduino microcontroller is the central hub driving the Go/No-Go sensory task, receiving analog input from the lick sensor and sending digital output to the host computer, water valve and physiological stimulator. The Ardunio monitors the animal’s response to the stimulus through a lick sensor which will ultimately determine whether the water valve is opened to deliver a reward.

The primary goal of this study was to establish an Arduino-based behavioral platform, which can be easily utilized for a variety of behavioral tasks with head-fixed rodents. The basic components of the platform for an operant conditioning behavioral task include: (1) a delivery system for water reward; (2) a lick detection sensor; and (3) a sensory stimulation apparatus. In our system (Figure 1), the Arduino microprocessor is the central hub which operates these components. The Arduino repeatedly monitors the signals from the lick sensor and controls the timing of water and stimulus delivery. As required, the Arduino sends a TTL trigger to other external devices to synchronize recordings and the animals’ behavior. A host computer regularly communicates with the Arduino via a serial connection to update the behavioral settings and readout the behavioral data logged in the Arduino. The source code for this system can be found online at https://github.com/palmerlab/behaviour_box, as well as additional documentation at https://palmerlab.github.io

### Behavioral Apparatus for Operant Conditioning

During operant conditioning, a subject’s operant behavior (in this case, lick response to a sensory stimulus) is reinforced by a reward. For head-fixed animals, the water reward is delivered through a waterspout located within reach of the animal’s mouth using a gravity flow water system. The reward delivery is controlled by a solenoid pinch valve (12 V DC, Takasago Fluidic Systems, Nagoya, Japan) and the volume delivered is determined by the duration of the valve opening and the height of the gravity water system. In our behavioral paradigm described below, mice receive approximately 10 μl water reward each correct trial.

Licking frequency is monitored using a custom-made piezo-based lick sensor. This consists of a piezoelectric wafer (0.6 mm Range Piezo Bender Actuator, PiezoDrive Pty Ltd, Callaghan, NSW, Australia) glued along the shaft of the waterspout (blunted 18-Gauge syringe needle). A tongue lick to the spout induces a small deformation of the wafer, which generates a voltage change inside the piezoelectric element. This signal is then amplified using a simple operational amplifier circuit (LM358N, Texas Instruments) to ensure the Ardunio microcontroller detects the lick-evoked voltage changes.

### Centralized Behavioral Control by an Arduino Microprocessor

**Figure 2:**
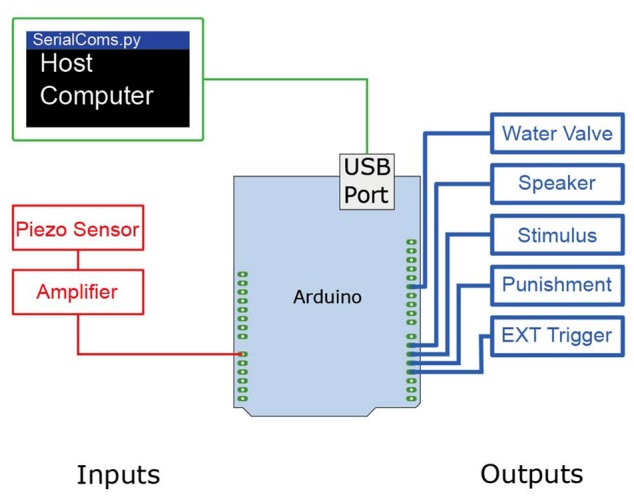
Wiring diagram of the Ardunio Uno Rev3 to control the Go/No-Go sensory task. The Ardunio is loaded with a controller program which controls various inputs (red) and outputs (blue). In brief, the Arduino controls the TTL pulses at the output pins, reads from the inbuilt analog to digital converter, and performs two way communications with a host computer. To operate the Go/No-Go sensory task, one analog input and four digital inputs/outputs are connected to the Arduino microprocessor. The signal from the lick sensor is amplified with a linear amplifier and the Arduino program thresholds the signal, counting rising edges as licks. When licks are detected in a *“*Go*”* condition the program sends a timed TTL pulse to a water valve to release a water reward to the lick port.

The Arduino Uno offers 13 digital input/output pins, as well as 6 analog to digital inputs with 4.8 mV resolution (0–5 V, 1024 bits; Figure 2). To operate the operant conditioning Go/No-Go task, the following analog and digital inputs/outputs are necessary (see Figure 2 for a schematic of the wiring diagram). *Analog inputs*: the amplified signal from the lick sensor is sent to an analog input pin. *Digital outputs*: four digital output pins are connected to: (1) sensory stimulator; (2) punishment consisting of a TTL-triggered valve gating a pressurized air line; (3) water valve; and (4) recording trigger.

The operant conditioning program running on the Arduino continuously reads the analog signal and detects individual lick events when the reported piezoelectric voltage signal crosses a given threshold. This sensing function runs in a loop, adding to a counting variable each time a lick is detected. The Arduino tracks the time and initiates the scheduled events for the task (e.g., sensory stimulation, opening the water valve, etc.).

### Data Transfer between the Arduino and Host Computer

The Arduino supports two-way serial port communications via an USB interface to a host computer. A Python script, (Python version 2.7.10) written around the pyserial library, is used to handle the sending and receiving of messages. The status of each experiment is continuously monitored and communicated between the Arduino serial port and the host computer. This requires the digital pins 0 and 1 to be unassigned in the Arduino code as these carry the relevant signals. The full set of global variables (listed in Table 1), excluding pin out assignments, is available for updating. In addition, during a trial, the status of each trial event is reported to the host computer. Successfully updating a variable results in the Arduino sending a message echoing the new variable and value to the serial port. In this way the system creates a running log of all settings and changes as they occur. All communications from the Arduino conform to yaml^1^ specification, with debug messages about trial events commented out. Yaml is a useful format for serialized data which emphasizes human readability. This self-documenting system was implemented because the stimulus timing and reward condition is updated on a trial-by-trial basis.

**Table 1:** List of variables communicated between the Arduino and host computer.

	Description	Units
**Variable inputs**
lickThres	Digital threshold to apply to lick sensor	converted 5 V/1024
mode	Sets the mode of the Arduino– habituation mode or operant mode	“o”/“h”
trialType	Code for the type of trial run	“G”/“N”
break_wrongChoice	Flag to end the trial early if a wrong decision is detected	0/1
break_on_early	Flag to cancel the trial if a lick is detected before stimulus onset	0/1
minlickCount	Number of licks required to trigger reward delivery, or punishment.	
t_noLickPer	Time prior to stimulus onset which must be void of licking before a trial is initiated.	ms
timeout	Amount of time to add to inter-trial interval if a wrong decision is made	ms
t_stimONSET	Time the stimulus is presented	ms
t_stimDUR	Duration of stimulus	ms
t_rewardDEL	Delay from the end of stimulus until activating the lick sensor	ms
t_rewardDUR	Duration the lick sensor is sampling for licks during response period	ms
waterVol	Amount of time to hold the water valve open for	ms
debounce	Duration the lick sensor needs to be high in order to call a lick (implements the simplest digital filter)	ms
**Outputs**
Water	Returns 1 if water was given this trial	0/1
N_timeouts	Returns the number of times the timeout was triggered since the end of the last trial
response	Returns the code for the response type	“h” (hit), “m” (miss), “f” (false alarm), “c” (correct rejection)
delta	Returns the difference in lick frequencies
pre_count	Number of licks made during the response period
post_count	Number of licks made during the baseline period
t_stimDUR	Returns the duration of stimulus	ms

The Python script is used to initiate each Go/No-Go task and therefore controls the timing of individual trials. After initialization, the Arduino defaults to a listening mode where it parses any variables that are transmitted. It will remain in this mode indefinitely until it receives the message “START” from the host computer, which triggers the initiation of a trial. This has the advantage of allowing the experimenter to pause the trials and make quick changes to the parameters without the need for touching the Arduino code, in turn avoiding recompiling.

### Running a Go/No-Go Sensory Task on the Arduino-Based Behavioral Platform

**Figure 3:**
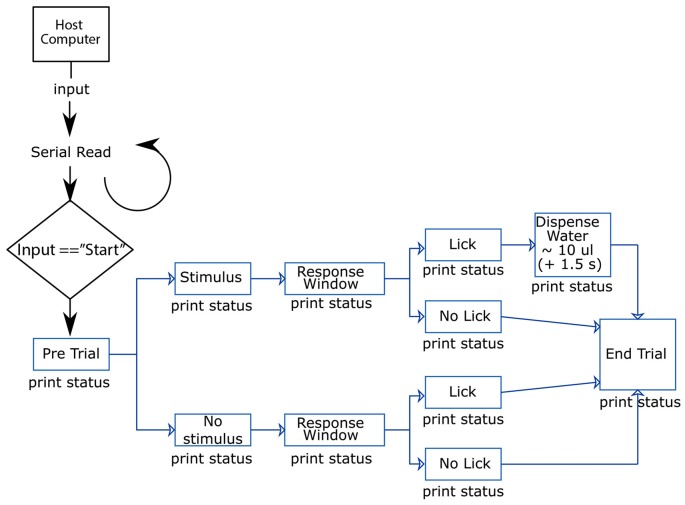
Flow chart illustrating the flow of information in-to and out-of the Ardunio. The host computer initiates a trial where either a *“*Go*”* or *“*No-Go*”* stimulus is randomly presented to the mouse. Only when the mouse correctly licks in response to the *“*Go*”* stimulus, they will receive a water reward.

The Arduino microcontroller is loaded with a custom written program^2^ which consists of two main functions; a basic *Habituation* mode, and a slightly more complex *Operant* mode. Here we discuss how to run these different behavioral training modes using the system described above (Figure 3). All procedures were approved by the Florey Institute of Neuroscience and Mental Health Animal Care and Ethics Committee and followed the guidelines of the Australian Code of Practice for the Care and Use of Animals for Scientific Purposes.

#### Habituation Mode

In the first training session the goal is to associate the “Go” sensory-stimulus with a reward. To achieve this, the habituation mode monitors the animal’s licking behavior and on detection of a lick, the sensory stimulus and water valve are triggered sequentially. This repeats until the mouse successfully associates the sensory-stimulus with a reward being delivered at the lick port.

#### Operant Mode

Once habituated to the sensory-stimulus and water reward, the mice are required to lick after the “Go” sensory-stimulus in order to get a water reward. This is controlled by the second main function in the Arduino code. This function starts a timer on the Arduino and if required, adds a specific trial delay. The recording trigger pin (digital output) is set high to initiate the recording systems. The controller then goes into a pre-stimulus delay period in which it detects licks and measures a baseline licking frequency. After this delay the stimulus is presented and a new lick count commences. A response period continues, and if the animal has licked during this time the water valve is opened and a reward is delivered. In the event of a lick during a No-Go trial, a punishment air puff can be delivered and a timeout period commences, during which additional licks delay the onset of the next trial.

### Two-Photon Imaging of Dendritic Activity during the Go/No-Go Sensory Task

The behavioral platform described here can be easily combined with various existing techniques/devices for recording neural activity as the Arduino processor can be used to trigger other software/hardware which support external trigger modes (EXT Trigger pin, Figure 2). Here, the EXT trigger sends a HIGH TTL signal at the start of a trial and remains on for the entire duration. The rising edge of this signal can be used to trigger episodic recordings, or the full signal may be used for devices that support variable length recordings. To prove the utility of this system, we performed two-photon calcium imaging from cortical pyramidal neuron dendrites while the animal was engaged in a reward-based sensory task. Mice expressing a genetically-encoded calcium indicator, GCaMP6f (AAV1.Syn.GCaMP6f.WPRE.SV40), in layer 2/3 pyramidal neurons in the primary somatosensory cortex were implanted with a chronic window. In brief, animals were anesthetized with isoflurane vapor (0.5% in 0.5 mL/min O_2_) during all surgery procedures and administered with lidocaine and meloxicam for local and general analgesia respectively. A small incision was made in the scalp, and using the stereotactic coordinates as a guide, a small craniotomy was made above the somatosensory cortex. The virus was delivered via a glass pipette, which had been backfilled with the virus aliquot and silicon hydraulic oil. A hydraulic piston was used to precisely deliver 100 nL of virus to the target site, 200–300 μm below the dura. After an incubation time of 7–10 days a head post and chronic window surgery was performed. Under isofluorane anesthesia (0.5% in 0.5 mL/min O_2_), the soft tissue of the scalp was removed. A small aluminum post was glued to the skull, using cyanoacrylate glue (Loctite 401, Henkel Australia Pty. Ltd., Sydney, VIC, Australia), and fixed firmly in place with C&B metabond® (Parkell Inc, Brentwood, NY, USA). A 3 mm circular craniotomy was made around the injection site, and a glass coverslip was fixed in place. The edges were sealed with cyanoacrylate glue, and dental cement (Jet Denture Repair, Lang Dental, Wheeling, IL, USA) was used to make a small well for the water-immersion lens (see Holtmaat et al., 2009).

**Figure 4:**
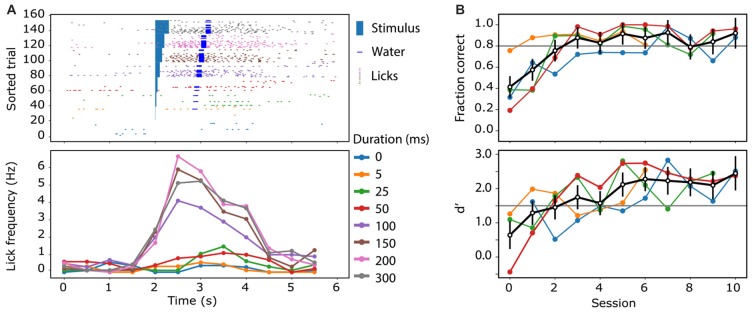
Measuring animal performance on the reward-based sensory perceptual task. **(A)** An example of the licking behavior of a mouse trained to report the presentation of a sensory stimulus (light blue rectangle) by licking. Trained mice increased their lick rate (colored ticks) dramatically after the stimulus (blue) and if the licking report was correct, water was dispensed (dark blue bar). **(B)** After habituation and operant training, mice learnt to associate a sensory stimulus with water reward on average (black) within three training sessions. Colored traces are the learning curve of individual mice (*n* = 4). The black trace illustrates the cohort average with standard error bars. The lower panel shows the signal detection sensitivity (d′) for the animals over the sessions.

**Figure 5:**
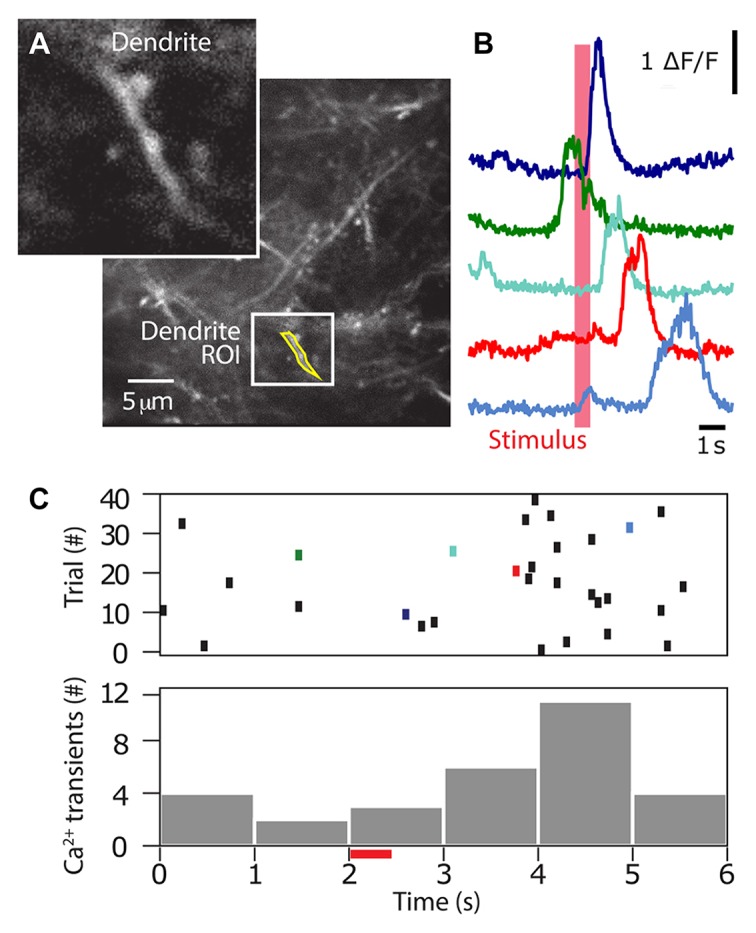
Two-photon calcium imaging during a sensory-based perceptual task. The Arduino based Go/No-Go behavioral task was performed simultaneously with two-photon calcium imaging. **(A)** Once trained (80% success), dendritic Ca^2+^ activity was imaged using two-photon microscopy through a chronically implanted window. In this example, Ca^2+^ transients (right) were recorded from the dendrite ROI (inset) on left. **(B)** Ca^2+^ activity throughout the behavioral task was reported for 40 trials for the dendrite shown in **(A)**. **(C)** Top, Ca^2+^ transients above a threshold (>3× standard deviation of the noise) are reported as ticks. Colored ticks correspond to colored traces in **(B)**. Bottom, summed histogram showing the number of Ca^2+^ transients occurring at different epochs throughout the trial. Stimulus was presented at 2 s (red bar).

After a recovery period of at least 3 days, the mice were acclimatized to head-fixation through several sessions of gradually increasing head restraint; beginning with momentary catch-and-release, and leading to 5–10 min restraints. During this time, and throughout the period of the experiment, animals had their access to water restricted to 1 mL/day. Their weight was monitored daily to ensure they did not drop below 80% of their pre-restriction mass. The animals were then introduced to the experimental rig, and trained to associate licking behavior with a reward, and stimulus, through the “habituation” mode described previously. Subsequently, they were trained to perform the Go/No-Go behavioral task as described above, in the “operant” mode. Here, the sensory stimulation was delivered to the contralateral forepaw using a small button (linear resonance actuator, precision microdrives, London, UK) and the ability of the mouse to detect the forepaw stimulation was tested by using stimulation of different durations (0, 5, 25, 50, 100, 150, 200 and 300 ms). Mice were able to reliably detect and report forepaw stimulation durations greater than 100 ms (Figure 4A). The great advantage of the Go/No Go behavioral paradigm over and above other complex reward-based behaviors is that it is rapidly leant. Mice typically reached a criterion of successful behavior (usually >80% “hit” and <20% false alarm rate, d′ > 1.5) after four sessions (Figure 4B). Using a two-photon microscope, we imaged the dendritic calcium activity of GCaMP6f-expressing layer 2/3 neuron dendrites in the forepaw area of the somatosensory cortex, while the animal was performing the Go/No-Go task (Figure 5A). Large Ca^2+^ transients occurred both spontaneously and during the Go/No-Go task in layer 2/3 pyramidal neuron dendrites (Figure 5B), with a greater number of large Ca^2+^ transients occurring after the initial stimulus delivery (Figure 5C). Using the behavioral platform to drive the sensory stimulation and behavioral recording enables direct correlation and association between neural activity and behavior, and opens the door to address many unanswered questions about the neural basis of behavior.

## Discussion

We describe an open source-based platform which can run a behavioral paradigm and deliver a reward based on the input received. This behavioral system is designed around the Arduino Uno Rev3 prototyping platform which controls TTL signals at the output pins, reads from the inbuilt analog to digital converter, and performs two way communications with a host computer. This Ardunio and Python based platform system is advantageous as it is a low cost, open source platform accessible to all. Our studies have focused on using this behavioral platform to deliver a sensory-based Go/No-Go paradigm (Figure 4) while simultaneously recording the associated neural activity using two-photon microscopy (Figure 5). Specifically, we record calcium activity in apical tuft dendrites during a sensory-based Go/No-Go task which can be used to investigate the synaptic feedback information conveyed to the primary sensory cortex. The described system can be used to control many behavioral paradigms.

Arduino based platforms have been developed to control many different apparatus’ involved in recording neural activity including an air-track system (Nashaat et al., 2016), two-photon imaging systems (Wilms and Häusser, 2015; Takahashi et al., 2016), treadmill displacement (Schneider et al., 2014), nose-poke trial logic (Wimmer et al., 2015) and Skinner box (Pineño, 2014). A similar system to the open-source Ardunio-based system we describe here has been developed previously to deliver liquid reward. The Rodent Operant Bucket (ROBucket) is an Arduino microcontroller-based platform which is used to control an operant conditioning chamber where mice are trained to respond for liquid reinforcers (Devarakonda et al., 2016). ROBucket controls two nose pokes, a drinking well, and a solenoid-controlled liquid delivery system. However, here we expand on this reward delivery platform, as we describe how the system can be controlled by the host computer to be modified on a per-trial basis according to the behavioral readout. Using a Python script, the system described here uses a host to ultimately control the progress of the behavioral experiment, adding delays or punishments where necessary. Other computer-based open-source systems have also been developed to control similar behavioral experiments. Bcontrol is a real-time linux/Matlab® software package for behavioral training developed by the Brody laboratory, Princeton University^3^; A. Mainen, C. Brody and C. Culianu). This system interacts rapidly with the experimental subjects and therefore provides high-time-resolution measurements of the behavioral events to enable coordination with other recording devices such as electrophysiology and imaging (Erlich et al., 2011; Brunton et al., 2013; O’Connor et al., 2013; Yang et al., 2016). More recently, Bpod is an open-source rodent behavior measurement and control system developed by Sanworks^4^. This custom-designed behavioral system is built around a finite state machine paradigm, where the transitions between states are dependent on the outcomes (behavioral measures) of the previous state. This allows precise control of stimulus delivery and behavioral measurements which can be synchronized with external recording devices (Pi et al., 2013).

Here we discuss how to set up an Ardunio and Python based platform system to control a Go/No-Go sensory behavior paradigm. Using an Arduino micro-controller and Python-based custom written program, a defined volume of reward water can be delivered to the animal depending on the timing and number of licks reported. We discuss the various components required to build a behavioral apparatus that can control and report a Go/No-Go sensory stimulus paradigm. This system enables the end user to control the behavioral testing in real-time and therefore it provides a strong custom-made platform for probing the neural basis of behavior.

## Author Contributions

AHM and NT designed the hardware and software. AHM and LMP conceptualized the manuscript. AHM, NT, MEL and LMP wrote the manuscript.

## Conflict of Interest Statement

The authors declare that the research was conducted in the absence of any commercial or financial relationships that could be construed as a potential conflict of interest. The reviewer NSD and handling Editor declared their shared affiliation, and the handling Editor states that the process nevertheless met the standards of a fair and objective review.

## Footnotes

^1^http://yaml.org/

^2^https://github.com/palmerlab/behaviour_box

^3^http://brodylab.princeton.edu/bcontrol

^4^https://github.com/sanworks/Bpod
